# Efficacy of the Endolysin-Based Antibacterial Gel for Treatment of Anaerobic Infection Caused by *Fusobacterium necrophorum*

**DOI:** 10.3390/antibiotics10101260

**Published:** 2021-10-16

**Authors:** Daria V. Vasina, Nataliia P. Antonova, Aleksei M. Vorobev, Aleksei I. Laishevtsev, Andrei V. Kapustin, Eldar R. Zulkarneev, Svetlana S. Bochkareva, Irina A. Kiseleva, Mariia N. Anurova, Andrei V. Aleshkin, Artem P. Tkachuk, Vladimir A. Gushchin

**Affiliations:** 1 N.F. Gamaleya National Research Centre for Epidemiology and Microbiology, Ministry of Health of the Russian Federation, 123098 Moscow, Russia; northernnatalia@gmail.com (N.P.A.); artem.p.tkachuk@gmail.com (A.P.T.); 2 G.N. Gabrichevsky Moscow Research Institute for Epidemiology and Microbiology, 125212 Moscow, Russia; vorobjew.alex2010@yandex.ru (A.M.V.); elzz89@mail.ru (E.R.Z.); cip1989@gmail.com (S.S.B.); irina6804@mail.ru (I.A.K.); andreialeshkin@googlemail.com (A.V.A.); 3 Federal State Budget Scientific Institution “Federal Scientific Centre VIEV” (FSC VIEV), 117218 Moscow, Russia; a.laishevtsev@gmail.com (A.I.L.); kapustin_andrei@mail.ru (A.V.K.); 4 Department of Pharmaceutical Technology, I.M. Sechenov First Moscow State Medical University (Sechenov University), 119435 Moscow, Russia; amn25@yandex.ru; 5 Department of Virology, Biological Faculty, Lomonosov Moscow State University, 119991 Moscow, Russia

**Keywords:** endolysin, *Fusobacterium necrophorum*, anaerobes, enzybiotics, gel formulation, topical treatment, animal model

## Abstract

Abscess formation is a common complication of severe life-threatening infections caused by obligate anaerobes. *Fusobacterium necrophorum* is among the frequently detected anaerobic pathogens from clinical specimens associated with liver abscesses, skin and soft tissue infections, or oral abscesses. The antimicrobial therapy for this kind of infection needs to be optimized. Here, we examined the possibility of treating *F. necrophorum*-induced abscess wound infections with candidate therapeutics based on three endolysins with activity against a broad spectrum of aerobe Gram-negative pathogens. Antibacterial gel containing three Gram-negative bacteria-targeting endolysins, LysAm24, LysAp22, and LysECD7, was formulated for topical use. Abscess formation was induced in rabbits with *F. necrophorum* and caused systemic infection. The survival and lifespan of the animals, general parameters, and biochemical and hematological blood tests were analyzed to assess the effectiveness of the gel treatment for the wound infection. The administration of the investigated gel twice per day for 5 days resulted in less acute inflammation, with decreased leukocytes and segmented neutrophils in the blood, retardation of infection progression, and an almost two-fold increase in the lifespan of the animals compared to the placebo group. The results indicate that endolysin-based therapy is an effective approach to treat anaerobic bacterial infections. The use of endolysins as independent pharmaceuticals, or their combination with antibiotics, could significantly reduce the development of complications in infectious diseases caused by sensitive bacterial species.

## 1. Introduction

Anaerobes represent normal human flora, which, however, could cause serious infectious diseases as a result of a breakdown of the mucocutaneous barrier or immunosuppression. Anaerobic organ infections caused by Gram-negative pathogens, such as *Bacteroides*, *Fusobacterium*, *Campylobacter*, and *Prevotella,* could promote brain abscesses, dental infections, and necrotizing infections of the skin and soft tissues, leading from local damage to life-threatening infections [1,2].

Abscess formation inside tissues and organs with localized necrotic lesions, during anaerobic infection, comprises a serious problem for treatment [3]. *Fusobacterium necrophorum*, a Gram-negative, non-spore-forming, strictly anaerobic bacterium in the Bacteroidaceae family, is a common bacterial isolate present in polymicrobial infections of affected tissues [4,5]. *F. necrophorum* is a causative agent of necrobacillosis, a severe disease that is characterized by inflammation and ulcerative or necrotic lesions, and is considered to be potentially fatal when not treated [6]. When involved in disease, *F. necrophorum* may cause localized lesions that spread via the bloodstream to internal tissues and viscera, causing necrosis [7]. Due to the anaerobic environment and antibiotic ineffectiveness at low pH levels inside the abscess, and the low availability of abscess lesions, antimicrobial therapy can be less effective and incapable of preventing generalized infection. Moreover, bacteria in deep tissue abscesses are embedded in biofilm-like matrices, comprising additional challenges for conventional antimicrobial chemotherapy [8]. This has led to the search for alternative solutions for anaerobic bacterial infection treatments, such as antimicrobial nanoparticles, immunotherapy, antisense RNA, microbiome-modifying therapies, and supramolecular biomaterials based on organized intermolecular self-assembly [9,10,11], as well as the employment of phages and phage-derived proteins.

Endolysins are lytic enzymes encoded by bacteriophages that cleave peptidoglycans in the cell walls of bacteria, and represent a potential alternative to traditional antibiotics [12]. Endolysins are active against a broad spectrum of strains [13], regardless of their antibiotic resistance status, as well as bacterial biofilms. Enzymes against Gram-negative bacteria have been widely studied in the development of antimicrobial agents to eliminate multidrug-resistant pathogens (*Pseudomonas*, *Klebsiella*, *Acinetobacter*, etc.), with an emphasis on bacterial respiratory pathogens [14] and against metabolically inactive persister cells [15]. Several in vivo studies describe the treatment of wound infections or bacteremia caused by multidrug-resistant nosocomial pathogens—*Klebsiella pneumoniae*, *Escherichia coli*, *Pseudomonas aeruginosa*, and *Acinetobacter baumannii* [16,17]. However, the potential of these enzymes for the treatment of infections caused by obligate anaerobic Gram-negative bacteria is obscure.

Here, we report the development of “GamaLysin CG01”, a complex antibacterial gel containing three phage endolysins, LysECD7, LysAm24, and LysAp22. CG01 is a candidate antimicrobial gel formulation based on phage endolysins with a broad spectrum of action against Gram-negative bacteria [13]. The efficacy of CG01 for topical treatment was investigated in a rabbit model of anaerobic wound infection accompanied by acute abscess formation. Wound infection and skin abscess formation were induced with *F. necrophorum* isolate, and survival, hematology, and blood clinical chemistry were assessed in groups treated with the developed therapeutic or a placebo formulated without endolysins. To the best of our knowledge, this is the first study demonstrating the in vivo efficacy and activity of phage endolysins against Gram-negative anaerobic bacteria using an *F. necrophorum* wound abscess model.

## 2. Results

### 2.1. Antimicrobial Activity of Endolysins against F. necrophorum

**Figure 1:**
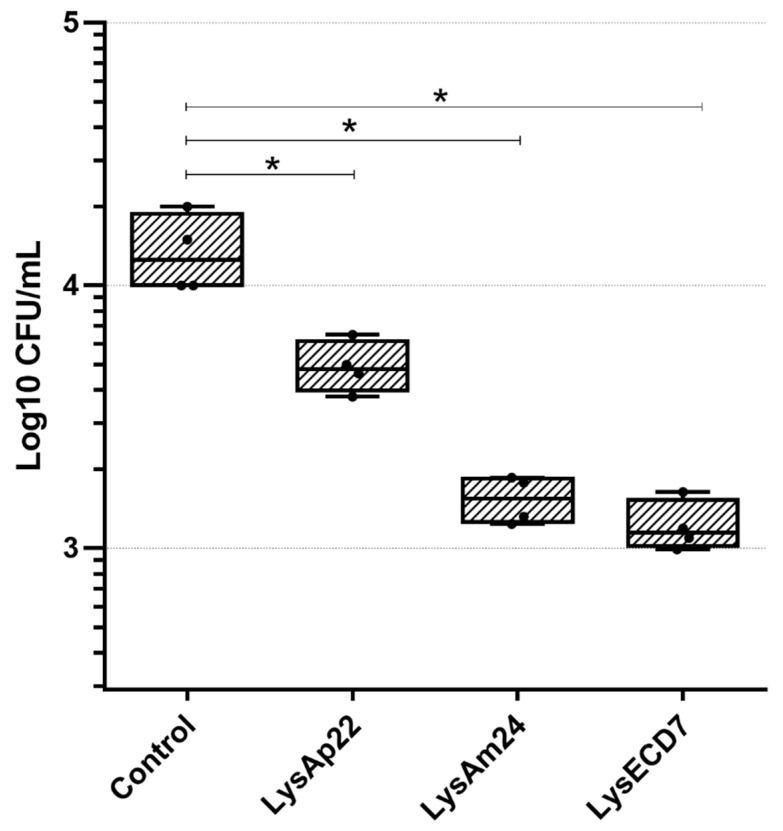
Endolysins activity against *F. necrophorum* 89-5 at a protein concentration of 100 μg/mL each. For all experiments, the mean values are shown with SD. An asterisk (*) indicates a significant difference in antimicrobial activity compared with the control with Tris-HCl incubation (*p* < 0.05, one-way ANOVA, Dunnett’s multiple comparisons test).

The three endolysins used as active pharmaceutical ingredients for the gel formulation provide a wide spectrum of activity against different Gram-negative bacteria, with lysozyme-like muramidase (LysAm24 and LysAp22) and endopeptidase (LysECD7) predicted activity [13,18]. However, the capability of LysECD7, LysAm24, and LysAp22 to reduce the bacterial load of obligate anaerobes was not previously studied. Thus, the antimicrobial activity of endolysins against *F. necrophorum* in vitro was assessed (Figure 1). Individual endolysins exhibited antimicrobial effects against *F. necrophorum*, reducing up to 1.25 × 10^4^ CFU/mL (1.05 log CFU/mL) in the case of the most active endolysin LysECD7.

The use of a mixture of three endolysins for the drug formulation was proposed to be more effective for bacterial cell elimination than individual enzymes, which can be attributed to the differences in enzyme substrate specificity and catalytic activity that target different peptidoglycan bonds in the carbohydrate backbone or polypeptide chains and interpeptide bridges. Thus, the gel formulation for topical treatment in a rabbit *F. necrophorum* infection model was fabricated using LysAm24, LysAp22, and LysECD7 at a concentration of 150 µg/g each, with Natrosol 250 HHX and PEG 1500 as excipients.

### 2.2. In Vivo Treatment Study and Model Description

**Figure 2:**
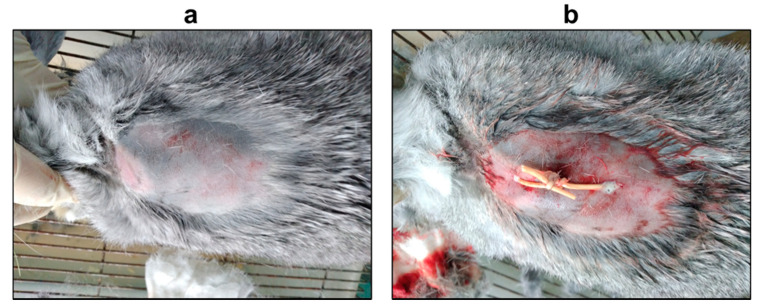
Withers abscess formation in rabbits caused by *F. necrophorum* (**a**) and animals with placed drainage (**b**).

The experimental animals were randomized into CG01 (*n* = 10) and placebo (*n* = 6) groups. According to ethical principles, an untreated animal group was not investigated in this study, as the used strain of *F. necrophorum* is shown to be highly aggressive, with a 100% mortality rate in rabbits when injected subcutaneously, in 10–14 days, in the absence of treatment. The abscesses at rabbits’ withers were induced with a subcutaneous injection of *F. necrophorum* suspension (3 × 10^9^ CFU/animal). Within 7 days, the abscesses had formed at the sites of injection of *F. necrophorum*. On the seventh day post-infection, the preformed abscess was lanced with two superimposed incisions, drained (Figure 2), and washed with saline to remove the exudate from the wound and ensure its outflow.

The test gel form was then administered, and 5 mL of CG01 was determined to be a sufficient volume for a single treatment of the abscess cavity. The treatment of the infected wounds lasted for 5 days, with two gel injections per day.

The survival and lifespan of the animals, general examination parameters (thermometry, assessment of behavior, general medical condition, and skin abscess area), and biochemical and hematological blood tests were analyzed for each group to assess the effectiveness of the gel treatment of the wound infection.

### 2.3. General Examination and Survival

The body temperature and skin abscess area were assessed before the infection and on the 7th, 9th and 12th days after the infection (Table 1).

On the seventh day, before the beginning of the treatment, the temperature of the animals in both groups had increased compared with the normal 38.3–39.5 °C [19], indicating inflammation. The body temperature of the animals in both groups was hyperthermic, although the CG01-treated animals had a lower average temperature on the 9th and 12th days after the infection (the 2nd and 5th days after the beginning of treatment) compared with the placebo group.

Despite the randomization in the CG01 group, the abscess area was significantly greater before the treatment (on the seventh day, before the beginning of the treatment) and during further experiments, which indicates more extensive wound formation in this group. However, the dynamics of the abscess area in the two groups were identical, and the growth reached 2.94 and 3.06 cm^2^ within five days of observation (the 12th day of study).

**Figure 3:**
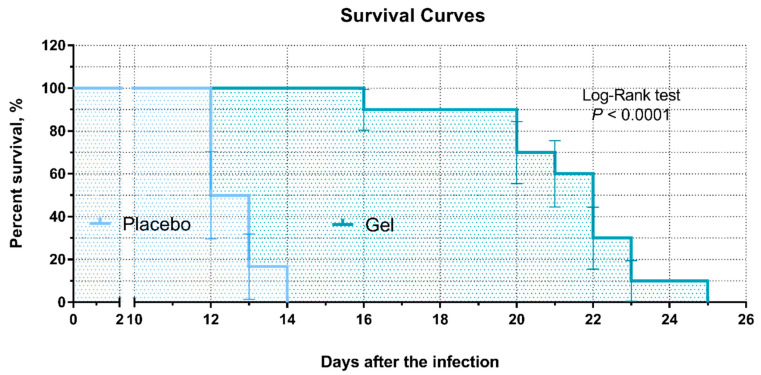
Survival plot of two studied groups. The log-rank (Mantel-Cox) test was used for analysis.

A significant difference was observed in the lifespan of the rabbits. The survival of the placebo and CG01 gel-treated groups showed a median survival of 12.5 days and 22.0 days, respectively (Figure 3). The maximum lifespan of the rabbits in the placebo group was 14 days, whereas it was 25 days in the CG01 group.

### 2.4. Necropsy and Bacteriologic Investigation

In all cases, immediate necropsy was carried out to confirm the cause of the animals’ death. Detected pathological changes were typical of rabbit necrobacillosis in a generalized form, so it was determined that the *F. necrophorum* abscess caused a systemic infection with secondary infection sites in the heart (hemorrhagic heart attack), liver (tension of Glisson’s capsule, liver abscess, liver necrosis), lungs (pneumonia), and biliary tract (cholestasis) (Table 2).

In addition to the pathological examination, necropsy material was taken (heart and liver) on the day of the animal’s death for bacteriological and mass spectrometry examination. *F. necrophorum* was isolated in 90% and 100% of cases from the heart and liver of fallen rabbits in the CG01 group, respectively, and in 100% of cases from both the heart and the liver in the placebo group, which confirms the establishment of the infectious process (Table 2 and Table 3).

Moreover, other bacterial species were found in the blood (*Prevotella oris*, *Propionibacterium acnes*) and liver (*P. oris*, *Clostridium cadaveris*, *Clostridium bifermentans*, *P. acnes*) of dead animals (Table 3). All the isolated species are commensal colonizers that are widespread at mucosal sites of the respiratory system, or are constituents of the gut microbiota [20,21]. However, their identification in heart and liver homogenates provides evidence of inflammatory disorders that can be caused by decreased immune responses due to severe infection.

### 2.5. Hematological and Biochemical Blood Tests

Blood samples at three time points (before the infection, and on the 11th and 20th days after infection) were analyzed for differential WBC counts and biochemical (ALT/GPT, AST/GOT, and total protein) parameters (Table 4). As no animals in the placebo group were alive by the 20th day after the infection, no data are provided for this group.

Biochemical parameters, such as AST and total protein, were within the normal range in both groups during the experiment. However, the ALT level was significantly increased in the CG01 group on the 20th day, indicating possible liver problems. However, the average level (on day 0, before infection) was initially higher in the CG01 group than in the placebo group. On the 11th day, there was a decrease in both groups, after which the ALT level in the gel group repeatedly increased beyond the reference range.

A hematologic analysis of white blood cell counts showed leukocytosis on the 11th day in the placebo group, indicating the development of acute inflammation, whereas leukocytosis was within the normal range in the CG01 group and significantly lower than that in the control, suggesting the efficacy of endolysin therapy. On the 20th day, the leukocytes in the CG01 group rose dramatically, which could be due to the termination of the treatment course on the 12th day and the generalization of infection. Additionally, the number of segmented neutrophils was significantly increased on the 11th day; however, this increase was almost two times lower in the treatment group than that in the vehicle gel group. On the 20th day, in the CG01 group, the segmented neutrophils were also above the reference range, which was assigned as a marker of microbial infection. However, the CG01 group showed a significantly lower value than the placebo group, implying more effective combat of the infectious process.

## 3. Discussion

The field of antimicrobial therapies based on phage-derived pharmaceuticals has actively developed, with in vitro and in vivo trials supporting the implementation of efficacious therapeutic products [22,23,24]. Several endolysins were tested against Gram-positive anaerobic *Clostridium perfringens*, the cause of life-threatening human infections such as enteric infections and gas gangrene. In vitro studies showed that the *Clostridium*-targeting endolysins CP25L and PlyGVE2CpCWB can reduce bacterial counts up to 5-log CFU/mL [25,26,27]. However, there are no described animal models of anaerobic infections treated by endolysins.

There are several studies about endolysin formulation strategies for topical or systemic use; for example, these strategies for endolysins topical application include the development of solutions, gels, nano-emulgels, nanoparticles, immobilization on bandages, or formulation in chitosan–protein sponges [28]. These studies mostly examine Gram-positive pathogen infections, while there is a lack of data on formulations of endolysins acting against Gram-negative bacteria.

*F. necrophorum* is a Gram-negative obligate anaerobic bacterium, an opportunistic pathogen causing numerous necrotic conditions. It is rare enough in clinical practice, but potentially life-threatening due to the possibility of bacteremia development. *F. necrophorum* strains with high toxin activity can induce mild infections or the death of animals [29], and, therefore, this is an appropriate species to model anaerobic infections in animals. The strain used in this study is known to cause the death of rabbits within 10–14 days after infection, due to metastatic necrosis in organs and tissues, which is accompanied by sepsis and septic shock [30]. Such infections often require an urgent surgical intervention, with subsequent debridement, and are not always effectively treated with bactericidal compounds [31].

Earlier, the in vitro activity and safety aspects of the *Myoviridae* bacteriophage endolysins LysAm24, LysECD7, and LysAm22 were investigated [13,32]. These enzymes are characterized by diverse domain organization (single-domain vs. two-domain) and different predicted mechanisms of action (lysozyme vs. peptidase activities). LysAm24 and LysAp22 are lysozyme-like endolysins, while LysECD7 is a Zn-dependent endopeptidase with predicted Ala-Ala peptidase activity. All of the assayed molecules are capable of lysing Gram-negative clinical isolates of *Klebsiella pneumoniae*, *Salmonella* sp., *Pseudomonas aeruginosa*, *Escherichia coli*, *Acinetobacter baumannii*, and *Enterobacter* sp., with a bacterial reduction of more than five orders of magnitude in concentrations up to 5 μg/mL of individual endolysins. LysAm24 and LysAp22 are also active against *S. haemolyticus* strains, but this activity is significantly less pronounced compared with the effect on Gram-negative pathogens.

The diverse effectiveness of endolysins against various types and genera of bacteria is known; therefore, complex preparations of enzyme mixtures are preferable. Since we expect that the sensitivity of different strains to the mixture will be higher than in the case of individual proteins, the gel was formulated with several phage endolysins with different spectra of action [13]. Thus, the multimode mechanism of action allows for profound cleavage of the peptidoglycan and decreases the chances of survival of bacteria.

Taking into account the literature data [16,17,33,34,35] and preliminary in vitro studies, we applied an average concentration of 150 μg/g of each endolysin, so the total protein concentration in the formulation was 450 μg/g. The proposed endolysin combination was formulated into GamaLysin CG01, containing hydroxyethylcellulose, and polyethylene glycol for optimal viscosity and adhesive properties, as they are widespread and non-toxic agents in pharmaceutical dosage forms, meet the requirements of regulators, and are approved for administration in many countries [36,37].

Here, we demonstrated the potential for the use of endolysin-based therapeutics for the treatment of necrobacillosis expressed as a decreased rate of infection spread and generalization in animals, which are the leading causes of death. This enabled an increase in the lifespan of infected animals of almost two-fold. Additionally, there were decreased amounts of leukocytes and segmented neutrophils in the experimental group, and their concentrations increased less and stayed within the normal range for a longer time than the control group. These data indicate less acute inflammation and a slower development of the infectious process after CG01 therapy. Thus, the data on skin abscess area dynamics, the animals’ lifespan, and hemological data support the efficacy of the gel.

Despite the 10-fold topical administration of the endolysin gel, all the animals died due to the aggravation of systemic inflammation and generalization of the wound infection process in both groups. The pathogens entered the bloodstream, causing general systemic necrobacillosis, confirmed by biochemical blood tests, as well as necropsy and mass spectrometry identification of *F. necrophorum* in the necropsy material. The increase in blood levels of aspartate and alanine aminotransferase is associated with extensive lesions of the gastrointestinal tract and liver in the animals, while total protein growth indicates tissue breakdown, which is characteristic of necrobacillosis infection [38,39].

In our case, antimicrobial therapy was initiated after abscess formation was well established. Thus, the initiation of therapy in this study is considered to reflect the treatment of the late phase of an anaerobic abscess [40], which resulted in 100% mortality of the animals in both groups. Thus, despite the significant retardation of necrobacillosis progression with the CG01 gel compared with the placebo, we propose that the full recovery of *F. necrophorum* infection may require a comprehensive treatment regimen using both local and systemic antimicrobial agents. In view of the clinical relevance, these results collectively show that endolysin-based antimicrobials may be efficacious in the treatment of a wound abscess infection that is induced by anaerobic bacteria, including *F. necrophorum*.

The endolysins used in this work initially did not possess the most pronounced in vitro efficacy against *F. necrophorum*. Despite this, the therapeutic efficacy of the antimicrobial gel increased the lifespan of the experimental rabbits compared with that of the rabbits treated with the placebo, by almost two times. This indicates that the search for new endolysins that are effective against this pathogen, as well as other sensitive Gram-negative anaerobic microorganisms, and the selection of optimal administration regimens of topical and systemic agents, can aid in the development of approaches to treat necrobacillosis and other bacterial infections.

## 4. Materials and Methods

### 4.1. Bacterial Strains

*Fusobacterium necrophorum* strain 89-5, maintained as a stock culture in the Collection of the Federal State Budget Scientific Institution “Federal Scientific Centre VIEV” (FSC VIEV), Russia, was used in this study. The cells were stored at –80 °C and cultured in appropriate medium at 37 °C prior to the experimental procedures.

### 4.2. Recombinant Expression and Purification of Proteins

Recombinant endolysins LysECD7 (NCBI AN: ASJ80195.1), LysAm24 (NCBI AN: APD20282.1), and LysAp22 (NCBI AN: CCH57765.1) fused to 8His-tag were obtained as described before [13]. Proteins initial coding sequences were artificially synthesized (Evrogen, Moscow, Russia), amplified from pAL-TA clones and integrated into the expression vector pET-42b(+) (Evrogen, Moscow, Russia), resulting in four pET42b-endolysin-8His plasmids. All constructs were sequenced via Sanger for errors. Recombinant proteins were expressed in *E. coli*, strain BL21(DE3) pLysS (chloramphenicol resistance) using induction with 1 mM β-D-1-thiogalactopyranoside at 37 °C for 3 h. Cells were harvested by centrifugation (6000× *g* for 10 min at 4 °C), incubated with lysis buffer (20 mM Tris HCl, 250 mM NaCl, and 0.1 mM EDTA, pH 8.0 and 100 µg/mL lysozyme) at RT for 30 min, and disrupted by sonication. The cell debris was removed by centrifugation (10,000× *g* for 30 min at 4 °C) and the supernatant was filtered through a 0.2 µm filter. Metal-chelate affinity chromatography with HisTrap FF column (GE Healthcare, Dornstadt, Germany) pre-charged with Ni^2+^ ions was used to purify the proteins on an NGC Discovery^™^ 10 FPLC system (Bio-Rad, Hercules, CA, USA). For this, the filtered cell lysate was mixed with 30 mM imidazole and 1 mM MgCl_2_ and loaded on the column pre-equilibrated with binding buffer (20 mM Tris HCl, 250 mM NaCl, and 30 mM imidazole, pH 8.0). The fractions were eluted with a linear gradient to 100% elution buffer (20 mM Tris HCl, 250 mM NaCl, and 500 mM imidazole, pH 8.0) and dialyzed against 20 mM Tris HCl (pH 7.5).

The purity of the proteins was determined by 16% SDS-PAGE and protein concentrations were measured using a spectrophotometer (Implen NanoPhotometer, IMPLEN, München, Germany) at 280 nm and calculated using predicted extinction coefficients (0.840, 0.831, and 1.4595 (mg/mL)^−^^1^ cm^−1^ for LysAm24, LysAp22, and LysECD7, respectively). All proteins were lyophilized and stored at −80 °C.

### 4.3. Antimicrobial Gel Formulation

An endolysin-based antibacterial composition was prepared in the form of a gel containing 150 μg/g of each LysAp22, LysAm24, and LysECD7, as well as Natrosol 250 HHX (hydroxyethylcellulose), 1% *w/w*, and PEG 1500 (polyethylene glycol), 1.5% *w/w*, as excipients. The gel base was mixed separately and sterilized at 120 °C for 15 min. The lyophilized endolysins were dissolved in 20 mM Tris-HCl buffer solution pH = 7.5 and added into sterile solutions of excipients to the appropriate concentrations. The gel base with Tris-HCl buffer without lysins was used as a placebo.

### 4.4. Antibacterial Activity of Endolysins In Vitro

Antibacterial assays were performed as described previously [18] with some modifications. To obtain a bacterial suspension of *F. necrophorum*, the culture was grown on Columbia agar (Conda, Torrejon de Ardoz (madrid), Spain) with the addition of 10% sterile defibrinated blood and incubated in a Bactron 300-2 anaerobic station (Sheldon Manufacturing Inc., Cornelius, OR, USA) at 37 °C in an atmosphere of 10% carbon dioxide, 10% hydrogen and 80% nitrogen for two days. The grown culture was washed with 1.5 mL of deoxygenated 20 mM Tris-HCl buffer at pH = 7.5 and resuspended until uniform turbidity. The suspension was diluted 100-fold in a suitable buffer to a final density of approximately 10^6^ cells/mL. One hundred microliters of the bacterial suspension and 100 μL of each endolysin at the concentration 200 μg/mL were mixed in 96-well plates, and a buffer without endolysins was used as the negative control. The mixtures were incubated at 37 °C for 30 min in an anaerobic station, and 100 μL was plated onto Columbia agar containing defibrinated blood. The number of surviving bacterial colonies was counted after 48 h of incubation at 37 °C in an anaerobic station. All experiments were performed in triplicate, and the antibacterial activity was expressed as follows: antibacterial activity (%) = 100% − (CFUexp/CFUcont) × 100%, where CFUexp is the number of bacterial colonies in the experimental culture plates, and CFUcont is the number of bacterial colonies in the control culture plates.

### 4.5. Animals and Housing

All in vivo studies were approved by the Ethics Committee of the Federal State Budget Scientific Institution “Federal Scientific Centre VIEV”, Moscow, Russia (approval No. 1091/22). Sixteen female Soviet chinchilla-breed rabbits (weight 2 kg, age 24–28 months) were used. Animals were purchased from Stolbovaya nursery for laboratory animals (Russia). All animals received care in accordance with the guidelines for accommodation and care of animals (ETS No. 123 “European Convention for the Protection of Vertebrate Animals used for Experimental and Other Scientific Purposes”). The animals were kept in a laboratory under veterinary observation in individual cages under normal conditions (room temperature of 22–24 °C; humidity of 30–70%), and a standard diet and water ad libitum were provided during the entire study.

### 4.6. Experimental Design and Treatment

**Figure 4:**
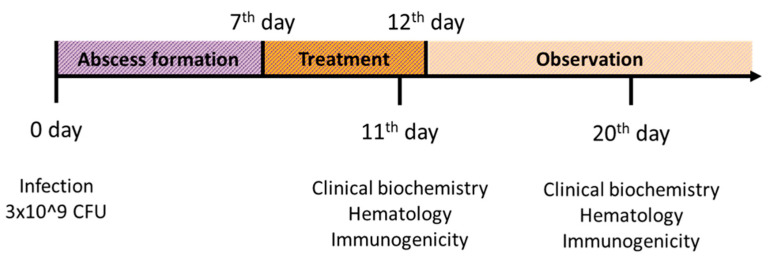
Experimental design of in vivo necrobacillosis wound healing study in rabbits.

The effects of treatment with endolysin-based gel were evaluated in the *Fusobacterium*-induced abscess model (Figure 4). An overnight suspension of the *F. necrophorum* strain growing in Kitt-Tarozzi nutrient medium at a concentration of 3 × 10^9^ microbial cells in a volume of 1 mL was injected subcutaneously into the rabbit withers. The accuracy of the manipulation was confirmed by the formation of an abscess at the injection site within 4–7 days. The planned observation period for the animals was 30 days or until their death.

Treatment with CG01 was started after the formation of an abscess in all animals on the 7th day. The experimental gel was injected into the animals after the abscess draining, installation of active drainage and ablution of the abscess cavity with sterile saline solution. To provide the outflow of exudate from the abscess, two incisions were made, so that one incision was below the localization of the second. During manipulations, passive type of drainage was used, which is explained by the shallow abscess localization in subcutaneous tissue. The operating field was pretreated with 70% medical alcohol and local anesthesia was carried out with a 0.5% novocaine (Grotex, St. Petersburg, Russia) solution in a volume of 0.1 mL at the site of incisions, and drainage was installed. Thus, for each incision, 2 injections (4 in total) at the distance of 2–2.5 cm were made. Prior to all surgical procedures, general anesthesia was also induced by inhalation of diethyl ether and maintained during surgery.

The drainage was installed lengthwise the longest plane of the abscess. The first incision of 0.5–0.7 mm in size was made following a surgical probe for drainage introduction into the formed cavity. The probe with drainage was brought to the most distant edge of the abscess, where the second incision was made, so that the drainage could be removed. After the drain was installed, it was tied in such a way that the animal could not untie and tear it off. After that, the abscess cavity was washed and the test gel was introduced into it. A volume of 5 mL gel was introduced into the wound cavity with a syringe and was enough to completely fill the wound. Wound irrigation and treatment with CG01 were carried out for 5 consecutive days twice daily. The placebo-treated rabbits (*n* = 6) received sterile gel free of endolysins at the same volume as that used for the CG01 group (*n* = 10). 

### 4.7. Efficacy Assessment of CG01 Gel

#### 4.7.1. General Examination

During the entire study, the animals were examined daily. The examination included thermometry, assessment of the behavior and general medical condition of the animals. The assessment comprised determination of habit, examination of visible mucous membranes, skin, and lymph nodes, measurement of body temperature, and systemic studies of the respiratory apparatus, cardiovascular system, digestive tract, urogenital and nervous systems.

#### 4.7.2. Pathological and Bacteriological Examination of the Animals

To confirm the cause of death of the animals, immediate necropsy was carried out. The pathological changes typical of necrobacillosis were determined, and infectious agents were isolated. Bacteriological examination of sectional material after the animals’ death was carried out as follows: 0.5–1 g of sectional material (liver, heart with blood) was inoculated into a tube containing Kitt-Tarozzi broth. Culture was carried out at 37 °C for 18–72 h. After the appearance of an initial bacterial biomass, the cultures were gram stained to assess the morphological and tinctorial properties. The cultures were transferred to blood agar and cultured under anaerobic conditions for 24–72 h at 37 °C, followed by identification of bacterial isolates using MALDI-TOF mass spectrometry. In brief, a single bacterial colony was resuspended in 150 μL of sterile deionized water. Then, 350 μL of 96% ethanol was added to each sample, which was vortexed and centrifuged for 2 min at 8000× *g* and 4 °C. The supernatant was removed, and the washing step was repeated twice. Forty microliters of a 70% formic acid solution were added to each sample. Subsequently, 40 μL of 99% acetonitrile was added and the solution was vortexed and centrifuged for 2 min at 8000× *g* and 4 °C. One microliter of each sample in triplicate was placed on a metal plate and left to dry in a sterile chamber at room temperature. Then, 1 μL of α-cyano-4-hydroxycinnamic acid (10 mg/mL) was applied to each spot of the sample and left to dry. The plate was analyzed with a Microflex MALDI-TOF mass spectrometer (Bruker, Karlsruhe, Germany) using the MALDI Biotyper 3.0 software package (Bruker, Germany). Scores above 2.0 implied reliable identification of the genus and probable species identification.

#### 4.7.3. Hematological and Biochemical Blood Tests

Blood was collected from the marginal ear vein at the following three time points: before the infection, and on the 11th and 20th days after infection. Hematological whole blood tests were performed on a Mindray BC-3200 hematology analyzer (Mindray, Mahwah, NJ, USA) and included differential WBC counts. Blood biochemical parameters were assessed with a ChemWell+ biochemical analyzer (Awareness Technology, Palm City, FL, USA) in venous blood serum without hemolysis. The following parameters were evaluated: alanine aminotransferase, aspartate aminotransferase (ALT/GPT, AST/GOT) and total protein. Prior to analysis, the blood was centrifuged for 6 min at 1500× *g* to obtain serum.

### 4.8. Statistical Analysis

GraphPad Prism 6 software (GraphPad Software Inc., San Diego, CA, USA) was used for all statistical analyses. Comparisons to the placebo group are reported by day with a significance level of α = 0.05. One-way ANOVA, Dunnett’s multiple comparisons test, and the Mann–Whitney U test were used to compare the data, and the χ^2^ test was used for comparison of categorical data; *p* < 0.05 denotes statistical significance.

## 5. Conclusions

In conclusion, we demonstrated that endolysin-based therapy is an effective, promising approach to treat anaerobic Gram-negative infections accompanied by abscess formation. We assume that the use of endolysins as independent pharmaceuticals, or their combination with antibiotics, could be advanced to recuperate other infectious diseases caused by sensitive bacterial species, and significantly reduce the morbidity and development of complications in infectious diseases of different etiology.

## Figures and Tables

**Table 1:** Body temperature and abscess area data in the two investigated groups.

Parameter	Days Post-Infection	Group	
		Placebo	CG01 Gel
Body temperature, °C	0 day	38.6 ± 0.8	38.6 ± 0.7
	7th day	**39.6 ± 0.7**	39.4 ± 0.5
	9th day	**40.7 ± 0.7**	39.7 ± 0.4 *
	12th day	**41.5 ± 0.4**	**40.5 ± 0.4**
Skin abscess area, cm^2^	0 day	-	-
	7th day	5.18 ± 3.97	12.88 ± 5.93 *
	9th day	6.65 ± 4.16	14.27 ± 6.09 *
	12th day	8.12 ± 5.16	15.94 ± 6.73

-—no data; *—significant difference between CG01 and placebo groups (*p* < 0.05). Hyperthermic values are shown in bold.

**Table 2:** The occurrence of pathological changes after infection in animals in the CG01 and placebo groups.

Secondary Infection Sites	Frequency, % (n)	
	Placebo	CG01 Gel
Liver abscess	50 (3)	70 (7)
Hemorrhagic heart attack	100 (6)	90 (9)
Cholestasis	100 (6)	90 (9)
Tension of the Glisson’s capsule	100 (6)	100 (10)
Liver necrosis	50 (3)	70 (7)
Pneumonia	50 (3)	80 (8)

**Table 3:** Results of mass spectrometry identification of anaerobic microorganisms in the necropsy material of rabbits.

Group	Microorganisms	
	Heart	Liver
Placebo		*F. necrophorum*
	*F. necrophorum*	*C. bifermentans*
		*P. acnes*
		*F. necrophorum*
CG01		*P. oris*
	*F. necrophorum*	*C. bifermentans*
	*P. oris*	*C. cadaveris*
		*P. acnes*

**Table 4:** Clinical chemistry and hematology test results from rabbits in the two investigated groups.

Test Parameter	Days Post-Infection	Placebo	CG01 Gel	Reference Range
**Biochemical Parameters**				
Aspartate aminotransferase (AST/GOT), IU/L	0 day	55.0 ± 12.3	66.2 ± 18.7	14–113
	11th day	45.3 ± 9.6	51.3 ± 16.2	
	20th day	-	56.2 ± 15.5	
Alanine aminotransferase (ALT/GPT), IU/L	0 day	69.9 ± 11.9	**84.0 ± 23.5**	
	11th day	56.7 ± 8.3	73.3 ± 16.8 *	48–80
	20th day	-	**87.5 ± 6.5**	
Total Protein, g/L	0 day	64.9 ± 5.7	67.0 ± 4.5	
	11th day	71.1 ± 6.6	75.2 ± 4.0	60–82
	20th day	-	**82.2 ± 2.2**	
**Hematology (Differential WBC Counts)**				
Leukocytes, 10^9^/L	0 day	7.28 ± 0.56	6.94 ± 0.84	
	11th day	**15.68 ± 2.64**	9.19 ± 0.87 *	5–13
	20th day	-	**14.96 ± 1.61**	
	0 day	0	0	
Myelocytes, 10^9^/L	11th day	0	0	0
	20th day	0	0	
Metamyelocytes, 10^9^/L	0 day	0	0	0
	11th day	0	0	
	20th day	0	0	
Banded neutrophils, 10^9^/L	0 day	0	0	0–0.2
	11th day	0	0	
	20th day	0	0	
Segmented neutrophils, 10^9^/L	0 day	1.57 ± 0.33	1.47 ± 0.34	1–4
	11th day	**13.67 ± 2.37**	6.85 ± 0.82 *	
	20th day	-	**11.77 ± 1.52**	
Eosinophils, 10^9^/L	0 day	0.4 ± 0.3	0.4 ± 0.2	0–1
	11th day	0.3 ± 0.2	0.4 ± 0.6	
	20th day	-	0.2 ± 0.1	
Monocytes, 10^9^/L	0 day	0	0.02 ± 0.04	0–0.5
	11th day	0.02 ± 0.04	0.20 ± 0.35	
	20th day	-	0.17 ± 0.30	
Basophils, 10^9^/L	0 day	0	0	0–0.5
	11th day	0	0	
	20th day	0	0	
Lymphocytes, 10^9^/L	0 day	5.3 ± 0.7	5.1 ± 0.8	3–10
	11th day	**1.7 ± 0.6**	**1.8 ± 0.5**	
	20th day	-	**2.6 ± 0.6**	

-—no data; *—significant difference between CG01 and placebo groups (*p* < 0.05). Values out of the reference range for rabbits are shown in bold.

## Data Availability

The datasets generated during and/or analyzed during the current study are available from the corresponding author on reasonable request.
